# Discovering anomalies in big data: a review focused on the application of metaheuristics and machine learning techniques

**DOI:** 10.3389/fdata.2023.1179625

**Published:** 2023-08-17

**Authors:** Claudia Cavallaro, Vincenzo Cutello, Mario Pavone, Francesco Zito

**Affiliations:** Department of Mathematics and Computer Science, University of Catania, Catania, Italy

**Keywords:** anomaly detection, machine learning, metaheuristics, classification, deep learning, recurrent neural network, fault detection, security threats

## Abstract

With the increase in available data from computer systems and their security threats, interest in anomaly detection has increased as well in recent years. The need to diagnose faults and cyberattacks has also focused scientific research on the automated classification of outliers in big data, as manual labeling is difficult in practice due to their huge volumes. The results obtained from data analysis can be used to generate alarms that anticipate anomalies and thus prevent system failures and attacks. Therefore, anomaly detection has the purpose of reducing maintenance costs as well as making decisions based on reports. During the last decade, the approaches proposed in the literature to classify unknown anomalies in log analysis, process analysis, and time series have been mainly based on machine learning and deep learning techniques. In this study, we provide an overview of current state-of-the-art methodologies, highlighting their advantages and disadvantages and the new challenges. In particular, we will see that there is no absolute best method, i.e., for any given dataset a different method may achieve the best result. Finally, we describe how the use of metaheuristics within machine learning algorithms makes it possible to have more robust and efficient tools.

## 1. Introduction

By anomalies, we mean values that deviate significantly from the distribution of a dataset or events that do not conform to an expected pattern. Anomalies can be caused by errors, system tampering, or novelties such as never-observed events. The detection of anomalies can be done by setting a decision threshold that allows an objective criterion to separate the outliers from the normal values, but on large volume data, this or the application of traditional methods becomes quite impractical if not impossible. Anomaly detection (AD) is applied in various fields such as log analysis, industrial control systems, diagnostic imaging, cybersecurity, and network monitoring. For instance, identifying medical anomalies allows for the provision of preventive treatments; the analysis of irregular images, videos, and audio has a great social impact because it also allows for the identification of identify fraud, illicit behavior, or fake users on social networks. In log analysis, where the goal is to find text that explains the nature and reason of the problem, the significant challenge comes from unstructured log data and messages differing in format. Creating a model based on historical data that identifies the normal behavior and/or values of a system allows for the establishment of a predictive maintenance program, the control or mitigation of threats and thus the reduction of financial losses due to fraud or failures. Anomalies can be identified through content-based, time-based, or connection-based analysis. Choosing the right model for the context and data to be analyzed presents a difficult challenge, as inefficient anomaly detection could generate false alarms or ignore problems, labeling them as normal behavior. In this work, we illustrate the advantages and limitations of each technique used in AD. Our review started from our research interests and, following keywords such as “*anomaly detection; intrusion detection; machine learning in anomaly detection; and metaheuristics in anomaly detection,”* we believe it covers the relevant literature, both in journals or conference proceedings, in a fairly adequate, balanced, and unbiased manner.

The paper is organized as follows: In Section 2, we will describe various machine learning methods, along with their variants, which are commonly used to detect fast or even in real time anomalies that could affect the proper functioning of an industrial, automotive, cyber, or space system; Section 3 briefly introduces some well-known metaheuristics and shows how they can be employed to optimize machine learning in choosing parameters and hyperparameters and in discovering anomalies in big data.

In Section 4 we will focus on one specific case study that shows a way to further improve the performance of existing machine learning methods for anomaly detection by using metaheuristic algorithms, and in particular the accuracy of a neural network for anomaly detection. Finally, in Section 5, the conclusions are presented and possible future research is addressed.

## 2. Machine learning and anomaly detection

Let us now briefly describe some of the machine learning methodologies applied to AD in different sectors.

We will start by reviewing some basic notions on neural networks. In a neural network, also called multilayer perceptron, the set of neurons or nodes that receive information from the outside represents the input level; the hidden levels are formed by internal neurons that share the same information of the same level and the output level at which neurons transfer already processed information. When the signal flow reaching the nodes of the network is multiplied by the respective weights, the weighted sum of each input signal is added to a threshold value called bias and evaluated by the non-linear activation function. Those usually used in artificial neural networks are the softmax function for the multi-label models and the sigmoid function for binary classification.

In more detail,

- the softmax function takes as input a vector of *n* real numbers and turns it into a probability distribution, i.e., *n* probability values proportional to the exponentials of the input numbers. In other words, before applying softmax, the vector components could be >1, or they might not sum to 1, or they might even be negative values. After applying softmax, each value will be in the interval (0, 1) and the sum of all values will be 1 and so that they can be interpreted as probabilities.
- the sigmoid function returns the weighted sum of inputs passed through an activation function and this output serves as an input to the next layer.

The training of the neural network serves to adjust the weights between the nodes and the bias so that the result provided is as close as possible to that of the ground truth. Therefore, the training phase is considered as an optimization problem (with the choice of suitable parameters), where we want to obtain the minimum value of a loss function. The appropriate hyperparameters to choose are concerned both with the structure of the neural network and with its training.

The architecture of a convolutional neural network (CNN) is formed by fully connected layers, pooling layers, and convolutional layers and its performance is influenced by the choice of number and size of these or other hyperparameters. Choosing them manually is expensive both in terms of time and hardware; therefore, methods such as the grid search, random search, and Bayesian optimization are used. The first requires a large number of combinations to test and evaluate the choice of hyperparameters, the second works with a random combination but it is suitable for small datasets, and the third performs well with a low number of dimensions.

### 2.1. Industrial control systems

In industrial control systems (ICS), the timely detection of anomalies in process dynamics requires strategies capable of signaling failures, attacks, or misconfigurations in real time and on a large scale. In fact, an anomaly in the physical components of an ICS could lead to unacceptable service interruptions or a risk to people's safety. Fortunately, the operation of an industrial plant follows a generally stable behavior as the data is correlated to the design specifications. Moreover, by using machine learning techniques and imposing spatial and temporal thresholds relating to the operation of the plants, we can limit the computational complexity. The experiments in Raman et al. (2021) were based on a real-time plant and a state of the art testbed called the Secure Water Treatment (SWaT). Sensor measurements were processed continuously and the reference model, which takes into account component aging and the performance lag of an ICS, was updated at regular intervals. In fact, the work showed that supervised learning algorithms, unlike unsupervised ones, based on historical data return a smaller number of false alarms, but on new data they fail to identify new anomalies. By testing various machine learning approaches on SWaT data, a semi-supervised detection technique based on multilayer perceptron (MLP) was found to be the best compromise.

In addition to the detection of anomalies, the control of industrial plants must consider the analysis of failures. It is difficult to recognize anomalies that have never occurred or to distinguish between different types of faults, especially when the working conditions are variable or the data is influenced by noise, which causes intraclass variance of the raw data and, in turn, degrades model performance. Manual extraction of failure characteristics is time consuming and subjective. This influences maintenance decisions, and relying only on previous knowledge of the plant is limiting. Huang et al. (2022) proposed a monitoring method that combines fault and anomaly detection with metric learning that avoids intraclass interference. The data are preprocessed by transforming them so to make them uniform in format and normalizing them to avoid large differences between them. The distance used to measure the similarity between the data is that of Mahalanobis (De Maesschalck et al., 2000) and, introducing the multicentric loss, features with small intraclass distances and large interclass distances are extracted. Features are mapped from a high-dimensional to a low-dimensional space, and the center of each found category is computed. Finally, the interclass loss, defined as the distance of each feature from the centers, and the interclass distance, the distance between centers, are taken into consideration and if the analyzed data belong to the known category of the training model, they are labeled as faults, otherwise as anomalies. In the training phase, the maximum distance from the centers is chosen as a decision-making threshold for identifying anomalies. The fault tracing module consists of a fully connected level and a softmax function that returns the probability that the input belongs to different features. Adam optimization algorithm (Kingma and Ba, 2015) was chosen in the learning process while the Silhouette score (de Amorim and Hennig, 2015) was used to evaluate the separation and cohesion between the clusters. Thanks to this, it was confirmed that multicentric loss gives better clusters, with similar data grouped together and well separated from those with different labels.

### 2.2. Industrial cyber-physical systems

Industrial cyber-physical systems (CPSs) allow the automation of large-scale production and distribution by connecting heterogeneous infrastructures together. Since remote access, cloud services, and applications used by CPSs are used in cyber-attacks, there are numerous vulnerabilities that can be exploited. Often, also because of an insufficient number of examples of high-quality attacks, the adopted security measures are poor and, therefore, intrusion detection schemes are necessary in this context. For this purpose, Li et al. (2021) developed DeepFed, based on a federated learning framework and a convolutional neural network with a gated recurrent unit (CNN-GRU). In this system, each industrial agent trains a local deep learning model on its own data (100 k entries with 26 parameters) and communicates with the cloud server, which through multiple iteration cycles learns the parameters for the common intrusion detection scheme and updates them. In the proposed Deep Learning model there is also a multilayer perceptron (MLP) module, consisting of two fully connected layers and a dropout layer that solves the overfitting problem. Finally, a softmax layer distributes the probability values of the predicted classes (different attacks or normal operations of the CPS). The performance of the proposed model was evaluated on various types of cyberthreats, showing that, with a reasonable number of communication cycles, the results in terms of accuracy and precision stabilize. DeepFed outperforms the local patterns of each agent and is also more effective than the state-of-the-art patterns of federated learning frameworks.

Among the CPS we mention, the modern automotive vehicles in which the hardware and software components are in communication with each other and an on-board computer must be monitored to identify anomalies. In the controller area network (CAN) used in programmable cars there is no authentication mechanisms and message encryption; therefore, an intrusion detection system (IDS) must be used to protect the network. An IDS system is responsible for monitoring traffic and alerting when anomalies such as known attacks or suspicious activity arise. CANnolo (Longari et al., 2021) is an IDS that analyzes CAN data streams during the training phase and detects anomalies using long short-term memory (LSTM)-autoencoders (Hochreiter and Schmidhuber, 1997; Yu et al., 2019). Autoencoders are unsupervised neural networks that compress input messages (and subsequently decode them), thus removing data noise and unnecessary information. During communication with a CAN, states, sensor values, and commands are transmitted. Although it is not possible to know the sender of the message, they are transmitted in a regular sequence, therefore they can be treated as a time series to be predicted. The dataset used to test this IDS included 10 million real-world packets retrieved from an Alfa Romeo Giulia Veloce. Malicious data differs from the ideal model created by LSTM and would be labeled as an anomaly. Through deep learning, the traffic sequence is in fact reconstructed, but using only legitimate data. To identify an anomaly, the reconstruction error between the real sequence and the modeled sequence is compared. An anomaly score, which represents the probability that an event is anomalous, is derived from the Mahalanobis distance between the observed data and the data in the model. CANnolo has been tested on a set of real urban and highway driving data of a car with an on-board computer (electronic control unit). To evaluate the system performance against real attacks, malicious messages are injected by sending commands, random data, ECU (Electronic Control Units) silencing, or repeating states previously transmitted. Unlike other CAN approaches, it does not require semantic knowledge of the data and no attack data has been used to train the detector, but it has the limitation of being relatively slow.

Another model for CAN packet intrusion detection was built by Boumiza and Braham (2019), using hidden Markov models (HMM). HMM have been applied in other fields such as speech recognition and computer vision and are effective for dealing with non-linear and time-varying systems. The attack hypothesis is that the attacker is able to access the network to know the IDS and the commands sent. Therefore, having knowledge of the regular communication flow and of the specific packets of the car model to be hacked, the attacker may modify the data field of the messages. The IDS system parameters are trained by extracting characteristics from the CAN messages and the constructed transition sequences are compared with the real data. The anomaly detection of the proposed IDS is in real time and, unlike signature-based detectors, it does not need many updates to identify already known attacks. Feature extraction from CAN packets occurs offline during training via the HMM architecture. It consists of hidden states and observable features and the transition between these is given by a probability distribution. The state transition from i to j is the probability of being in state i at time t and transitioning into state j at time t+1. By setting a decision threshold, if the real flow misaligns beyond this value with respect to the reconstructed sequence, the analyzed data is identified as an anomaly. In fact, each ID is responsible for delivering certain types of messages and, when the mode of action changes, the generated packets are probably illegitimate. To test this approach, multiple CAN packets recorded on a real vehicle for 24 h over several months were evaluated. The proposed IDS shows good performance with a higher detection rate than deep neural networks (DNN) and returns a lower number of false alarms in the same comparison. If we increase the number of parameters of the HMM, i.e., the number of mode values, the performance gets worse, and this is the limitation of this method.

### 2.3. Machine learning algorithms and IDS

In Saranya et al. (2020), we see the comparison of the performances of three supervised machine learning algorithms to detect intrusions on simulations of a military network (KDD Cup 1999 dataset, Lee et al., 1999). It is observed that the results depend on the nature and size of the data, and that in the case studied the random forest (RF, in Li et al., 2016) algorithm outperformed linear discriminant analysis (LDA) and classification regression trees (CART, in Breiman, 1984). The more trees present in the model, the higher the accuracy of RF, although it increases the probability of overfitting.

Tait et al. (2021) compared six ML methods applied to the UNSW-NB15 (Moustafa and Slay, 2015) and CICIDS2017 (Sharafaldin et al., 2018) datasets and showed that in multiclass classification (different attack types and benign traffic), k-nearest neighbor (K-NN) gives more accurate results, while in binary classification (intrusions and normal values), RF provides higher accuracy scores. All the techniques used are supervised, except the k-means, which is an unsupervised clustering algorithm and misclassified almost half of the data (binary classification) and a third with multiclass labels, thus being considered the worst approach. The datasets were large in volume and required a reduction of the attributes based on the evaluation of the information gain through the Weka tool: the advantage lies in the impact on the computational power of the ML algorithms on the preprocessed data.

A network intrusion detection system can generate alerts based on signatures, anomalies, or a combination of the two. The first is a static analysis, limited to known intrusions; the second is based on the normally observed traffic. Song et al. (2020) presented a deep learning-based IDS of network traffic via a deep convolutional neural network (DCNN). During the training phase, the labeled CAN messages are exploited for offline supervised learning while the real communication flow is examined in the detection phase by classifying the messages into two classes: normal or anomalous. The structure of the Inception-ResNet model was used, but the size and levels of the DCNN were reduced in order not to delay message collection and attack detection. This system protects against attacks such as injections of manipulated messages, but does not overload the network as happens when using signatures with secret keys. In addition, training is less expensive than a deep belief network-based model (DBN) and the proposed IDS has significant detection performance.

Saboori et al. (2010) showed an application of *Apriori* (Agrawal et al., 1993), a data mining algorithm, to generate meaningful relationships between available data and network attacks. In this way, it is possible to make predictions of the type “if there is an antecedent, then there is a consequent with a certain probability.” These are called association rules and can be used to check for antecedent-consequent implications for coexisting anomalies in at least a fixed percentage of the data. *Apriori* represents an advantageous method as it is able to extract frequent patterns from large volumes, which would be impractical to identify with brute force methods and using distance metrics for comparisons.

### 2.4. AD and urban traffic

Anomaly detection can also be applied in urban informatics. The approaches used in the literature for the detection of traffic anomalies are divided into clustering, classification with supervised and unsupervised learning, and statistical detection algorithms based on the nearest neighbor. In Zhu et al. (2018), London vehicular data was analyzed so as to evaluate an incident prediction method. Traffic can be divided into recurring and non-recurring; the former is linked to congestion at peak times and weekends, and the latter is classified as an anomaly linked to accidents, dangers from adverse weather conditions, or unforeseen works. Vehicular flows were recorded by inductive loop detectors (ILD), and contained recurring traffic and unplanned incidents during the study period from 1st January 2015 to 24th March 2015. The aim of predicting non-recurring traffic as accidents is to mitigate vehicular congestion, obtaining economic, and environmental benefits for road drivers, as well as avoiding further negative impacts on safety. It also makes it possible to manage traffic to quickly restore regular flow. While other works in the literature are limited to predicting accidents in specific straight sections such as tunnels or highways, the described method pays attention to complex road networks.

Anomaly detection has also been applied to video and object recognition so as to tag anomalous trajectories, such as u-turns, unusual stops, and driving in the wrong direction. A framework named ELM-HTM was presented in Sekh et al. (2020) for the unsupervised classification of time series based on extreme machine learning (ELM, in Huang et al., 2012) and hierarchical temporal memory (HTM, in Wu et al., 2018). The latter learns the low-level characteristics of the model, and the former the high-level similarities. Key challenges in this context include the noise and changing nature of data in the real world, the limited availability of labeled data, and the necessity for the amount of learning time to be small enough so to apply the online method. ELM is a very simple model that requires little data and takes less time to learn than traditional deep learning methods. It has the disadvantage of assigning random values to the weights of the hidden layers. To avoid this, statistical weights are assigned from probability distributions using the restricted Boltzmann machine (RBM, in Pacheco et al., 2018). HTM has a structure made up of local context, feedback, and flashfoward, and transforms the input pattern into spatio-temporal mini-columns with reduced data. A strong feature of HTM is that it can be trained in real time by inputting the transformed sequences into sparse distributed representations (SDR). Subsequently, a spatial pooler (SP, in Cui et al., 2017) is applied to reduce the complexity of the trajectory, and the DBSCAN algorithm (Ester et al., 1996) is also applied to set the number of hidden levels of ELM equal to the number of obtained clusters. The highest level of the architecture is softmax which returns the index of normality; the similarity of the space-time sequences is evaluated with the overlap with the model. Normality distance is determined through DTW barycenter averaging (DBA, in Petitjean et al., 2011). Finally, the sequences of anomalous points are classified as anomalous trajectories in the output and a normality score is associated to the sequences that fall within a chosen threshold. Evaluating the obtained results, it is highlighted that the training time is significantly shorter than that for LS-SVM (Chen and Lee, 2015), ELM, HTM, and LSTM, even if the number of samples increases.

Short text messages from the social media Twitter were used by Zhang et al. (2018) to detect anomalies in road traffic using a deep learning approach. The time frame under investigation, 1 year, included about 3 million tweets geolocated in the New York and Northern Virginia areas and a list of incidents published by the Department of Transportation was used as ground truth. Since the data was noisy, text mining methods were applied to extract key terms related to traffic accidents, called tokens. From a collection of 100 newspaper articles discussing accidents, the most frequent words describing them were selected and then candidate tweets containing these terms were extracted. Some texts may contain grammatical errors, therefore terms similar to the keywords were checked and tagged manually. Furthermore, all derived words were transformed into their common linguistic root. Regarding the spatial location and the date and time of the tweets, a space-time tolerance was set as the incidents took 1–2 h to resolve and users may have published their posts after having moved a few miles away from the incident. After mining tokens in candidate tweets, a deep belief network (DBN) was applied and the results compared to other supervised deep learning methods. Furthermore, to capture relationships between different tokens, the associations rules of the *Apriori* algorithm were extracted, showing that the accuracy in detection was thus improved. When comparing the findings with the official incident register, the accuracy obtained from the tweets was 85%, which exceeds that of SVM, LSTM, and supervised latent Dirichlet allocation (sLDA). The performance further improved when the number of tokens in the used model was increased. More clusters of tweets in the same area led to the higher likelihood of true positives. As for false positives, they could be due to geolocation errors. For minor accidents that did not require the intervention of the traffic police, it emerged that 80% of the tweets talking about accidents could be linked to these anomalies even if they did not appear in the official register. Twitter's textual data are effective due to their timeliness, but they cannot constitute a main source for detection, rather they are to be integrated with other traditional ones.

### 2.5. AD and crowd modeling

Cavallaro et al. (2020) proposed a multi-agent system aimed at travel planning to recommend, according to preferences, highly visited places, or avoid crowds. A probability algorithm estimates the number of people who move around the city, staying for a significant time in areas called staypoints (SPs). The collaboration system is based on the choices of other users (collaborative filtering) and is supported by a large amount of data, sent to a server that processes the occupied positions. It was tested on the trajectories formed by a total of 11 million GPS points, and the routes are divided into six daily time slots in order to characterize the movements and stops of people at different times of the day. Starting with the map of San Francisco, the space was divided through a grid of equal square cells, and the SPs are projected onto these. The greater the number of SPs falling into the same cell, the more crowded it will be considered. The algorithm labels cells with a high probability of visiting and large crowding (greater at a fixed density) with 1 and those a low probability of visiting with 0. The estimate is based on the co-occurrence of places (and predictable routes between SPs) highly frequented by a number of people exceeding a threshold. The application that acts as an agent tracks the movements of the users and has the purpose of alerting the visitor when the preferred destinations are overcrowded. After having identified the trajectory flows (i.e., spatio-temporal sequences of different vehicles) common to a considerable number of users and the relative destinations, these are compared with the overcrowded SPs. If a considerable number of users go to an area not identified as an SP and stop there, the flow in question will be considered an anomaly as it deviates from the system's forecast.

The analysis of the images for the identification of anomalous behaviors and for the estimation of the crowd density is extremely important in video surveillance because it allows the possibility to alert human operators and to design public spaces respectively. Chaker et al. (2017) developed an unsupervised method for anomaly localization in crowd analysis using a social network model (SNM), which is represented as a graph in which the nodes are people and the links are the social relationships between individuals. The video frames are divided into space-time cuboids, with a partitioning that is proportional to the density of the crowd, and a local social network (LSN) model is created in each of them. Spatially close nodes, within a fixed threshold, form a connected component for the graph. To identify local behaviors, similar characteristics such as direction and amplitude of motion are grouped through cosine similarity, and dynamic time warping (DTW) is used to measure velocity similarity. For each scene, the results are stored in similarity and adjacency matrices. Finally, the global crowd behavior is modeled with a global social network (GSN), in which Hierarchical agglomerative clustering unites similar LSNs from different cuboids in a time window. Using the hierarchical partitioning scheme, some lower level LSNs with few nodes marked as abnormal can be merged with other higher level LSNs. Finally, global anomalies such as those small and isolated social networks are detected and localized in the video scenes. The approach was tested on two video sequences generated by fixed cameras and the results of the SNM were compared with state-of-the-art methods. In conclusion, it is shown that other models such as the social force model (SF, in Mehran et al., 2009), mixture of dynamic texture (DTM, in Mahadevan et al., 2010), or the mixture of optical flow (MPPCA, in Kim and Grauman, 2009) have a lower accuracy in the detection and localization of crowd anomalies than SNM.

Cavallaro and Vizzari (2022) presented a different approach to spatio-temporal analysis for the detection of groups of pedestrians within a large data set. The movements are acquired by sensors in a shopping center and, through an algorithm, the hesitation points, which characterize the cadence of the pedestrian path, are identified. Quickbundles (QB), an unsupervised clustering algorithm used in tractography to bundle brain fibers, is used to identify groups of pedestrians moving together throughout their path. QB efficiently groups large quantities of trajectories into pedestrian flows according to their proximity and angle of movement (spatial similarity criterion). A subsequent phase of the proposed algorithm instead filters the identified groups moving together according to a temporal similarity criterion. The anomalies highlighted by the results are of two types: groups of pedestrians (clusters) which exceed a reasonable density of individuals for the corridors under examination (overcrowded areas) and spatio-temporal trajectories similar to the identified clusters but not included in these because they are interrupted or segmented, as they are no longer detected by sensors by mistake. Crowd analysis is effectively managed by QB while having minimal assumptions about the nature of the groups and the space and supports the architectural design of the interior environments.

### 2.6. AD and social networks

Anomaly detection also finds application in social networks, in order to guarantee the security and privacy of users. However, the growth of data represents a clear challenge. Anomalous online activities include spreading fake news, spam, phishing, harassing messages, and unusual/atypical behavior. Their experiments were performed on a synthetic dataset of 20 k users and on 10 k (real) Facebook profiles. The growing phenomenon of cyberbullying leads vulnerable teenagers to extreme acts, including suicide. DT-SVMNB uses the decision tree (C5.0), support vector machine (SVM), and naive Bayesian classifier (NBC) for the classification of depressed or suicidal users in social networks. Initially, characteristics associated with class labels are identified, based on the user's profile (such as number of friends, followers, and posts) and the content of the messages. In the first phase, the C5.0 makes a distinction between normal and anomalous users from the data set of social networks and the training uses synthetic and real data. This method is advantageous because it requires less memory than the other algorithms and allows you to remove irrelevant attributes from the data. In the second step, the SVM classifies the anomalous users identified in the previous phase into happy and disappointed users. In the last phase, the NBC extracts suicidal users from the “disappointed users” subset on the basis of a dictionary. The proposed approach had an accuracy of 98% on the dataset used. Furthermore, the system provided a higher level of accuracy in the detection of suicidal users compared to other existing methods.

### 2.7. AD and climate events

Large-scale climate simulations are of great importance as they can be used for risk management and climate change mitigation. Racah et al. (2017) have provided a public dataset, ExtremeWeather, which collects a simulation of extreme climate and weather events from 1979 to 2005. Through a temporal resolution of 3 h, more than 78 k images are available. These multivariate data are used for training semi-supervised models, in order to detect and localize four extreme climatic events: tropical cyclones, tropical depressions, extratropical cyclones, and atmospheric rivers. The ground truth labels relating to the four types of meteorological anomalies were derived from the Toolkit for Extreme Climate Analysis (TECA) application. As input, 16-channel climatic images were provided for each time slot, representing the 16 climatic variables such as pressure, temperature, precipitation, wind, cloud fraction, and water vapor. The model was trained with Adam. The authors implemented a 3D convolutional encoder-decoder (on dimensions height, width, and time), showing its significantly better performance than a 2D encoder-decoder (height and width). It is used for the reconstruction of unlabeled data. Good and reliable predictions have been made on the areas where extreme weather events can occur.

### 2.8. AD and log analysis

The CNAF (National Center of Information Technologies) hosts the Italian Tier-1 center, one of the eleven Tier-1 centers of the Worldwide Large Hadron Collider (LHC) Computing Grid. It is the IT department of the National Institute of Nuclear Physics (INFN) that provides and maintains the computing infrastructure. In this datacenter, a large amount of data for high-energy experiments is securely stored and the CNAF provides support to the LHC experiments at CERN in Geneva. The status of services used by users is stored in specific log files, in which it is difficult for runtime properties to be understood by manual analysis. Therefore, it is necessary to automatically diagnose problems that interfere with the proper functioning of the data center, detecting anomalies in the log files based on the messages in order to implement solutions. Identifying machine behavior patterns allows for predictive maintenance, so as to act promptly and reduce breakdowns and downtime. The analysis of the logs for the anomaly detection at the CNAF was performed by Cavallaro and Ronchieri (2021) through the invariant mining model and NLP techniques that do not require any knowledge of the data. These approaches have been combined for a scan of Tier-1 log messages to help system administrators understand the health of services and code anomalies. The challenge in this type of AD is due to the heterogeneity of the logs that are produced; moreover, they are semi-structured texts with timestamps that grow in size as the operations of the machines increase. The log files are open for writing and contain the date and time of the service activities (with an average of 120 k lines for a daily summary), but each software or application can use a different keyword to refer to an anomaly. Through an NLP approach to this work, the logs are preprocessed, such as dictionary-based log compression and the removal of unwanted characters. In addition, variables to be included for learning are selected, such as date, time, hostname, Internet Protocol (IP) address, service name, process identifier, component name, and message. Through word2vec (Mikolov et al., 2013), the keywords are mapped in space and the vectors that represent them are compared to each other through the Euclidean distance. The goal is to produce distance-based clusters to group and classify the normal and anomalous events recorded by the logs. Invariant mining is a machine learning model that is not based on the nature of the data but that automatically highlights the breaking of invariants. In a process, the opening of a file must correspond to its closing, or the number of jobs of the same type started and finished must coincide. The input of the algorithm is an event counting matrix, built starting from the logs that expresses the relationships between messages and originating hosts. The output returns 1 or 0 depending on whether there is an anomaly or not, respectively, in the message for a specific event, and this allows the tracing of the hosts and the causes that triggered the errors. The adopted techniques start from the conditions of the systems with off-line monitoring and allow the prediction of future states and the generation of alarms in real time when the same anomalous messages occur. The results showed an F-measure above 86% in the detection of unusual activity at Tier-1; thus, the approach is promising for short and long term management.

The CNAF is one of the primary contributors of the EU-founded IoTwins project and created a big data platform (BDP, in Tisbeni et al., 2021) for optimized and replicable industrial and facility management models. It focuses on the monitoring of IoT/Edge/Cloud integrated infrastructure and on predictive analysis of faults and support on troubleshooting. The CNAF's alarm system is based on Sensu and Slack. BDP is important for the detection and the analysis of heterogeneous data from different sources. It is an infrastructure as service that can be accessed to perform the analysis of anomalies. BDP deals with the management of different resources and different technologies has been developed for the monitoring system to control the status of services at CNAF. BDP is a reliable, extensible, scalable, and manageable platform for the collection and analysis of big data to be offered as a service. It is a cloud-native architecture, that can be federated with other clusters. The users are able to visualize the data in a simple way through an interface. The anomaly detection at CNAF greatly benefits from BDP; through the performed clusterization, it is possible to perform the analysis in streaming to label the new entries as they are included in the database.

## 3. Metaheuristics, machine learning, and anomaly detection

We will now briefly introduce some well-known metaheuristics and discuss their use in discovering anomalies and the optimization of machine learning methodologies.

In several of the examples we will overview, we will refer to the NSL-KDD dataset (Tavallaee et al., 2009), which is an improved version of the KDD CUP 99 dataset for intrusion detection. The dataset comprises various types of attacks that can compromise the security of a computer network by unauthorized individuals. It consists of 41 features, 125, 973 records in the training set, and 22, 544 records in the test set. The attack classes are divided into four different categories: U2R (User to Root), R2L (Remote to Local), Probe (surveillance), and DoS (Denial of Service).

### 3.1. Evolutionary computation

By evolutionary computation, we mean a class of algorithms inspired by biological evolution. Typically, they have a population-based trial and error stochastic metaheuristic (see Fogel, 2006).

In evolutionary computation, an initial population (i.e., a set of candidate solutions) is randomly generated and iteratively updated. Each new generation is produced by stochastically selecting some good solutions and removing some bad ones. In other words, the population of solutions is subjected to natural selection and mutation. The most widely known examples of evolutionary algorithms are: genetic algorithms (GA, see Goldberg, 1989), artificial immune systems (see Castro and Timmis, 2002), and differential evolution (see Storn and Price, 1997). Most common problems faced when designing a population-based optimization algorithm are related to the evolutionary operators which are used and the way the parameters are experimentally fixed, such as the number of generations a particular solution stays in the population (see, for instance, Di Stefano et al., 2016; Vitale et al., 2019).

#### 3.1.1. Genetic algorithms

Genetic algorithms (GA) are well-known search algorithms which are used to find approximate solutions to hard computational problems, often very close to the optimal one. Thanks to their key features, i.e., working with a population of solutions, the ability to exploit the information discovered during the evolution, the combination of random and deterministic rules, the recombination of good solutions to create, statistically, a population of better solutions (as guaranteed by the schema theorem), and many others, GAs are well-suited to efficiently explore the search space, especially when it is of large size. As experimentally verified, GA's often quickly converge toward approximate and acceptable solutions. It is well-known and documented in the literature that genetic algorithms and any population-based metaheuristic in general work well when dealing with very large instances of hard problems, that is, those instances where exact polynomial-time approximation algorithms become, in practice, too slow. GA's and metaheuristics in general are furthermore widely used in real-time optimization problems, where time constraints are crucial, because they are able to significantly reduce the search time. For more details, see Talbi (2009).

In the first example of the use of GA described here, combined with neural networks, we can see how we can manage to determine the optimal solution in choosing the hyperparameters in a limited number of iterations or try to converge to the global optimum as fast as possible. Furthermore, these algorithms are also easily deployable and parallelizable. Muhuri et al. (2020) presented an IDS to classify the NSL-KDD dataset, through the combination of the GA metaheuristics with deep learning. In particular, GA was combined for the optimal feature selection in a long short-term memory-recurrent neural network (LSTM-RNN) model. The hyperparameters set in the experiments concern the batch size, epoch, learning rate, dropout, and activation function. A fitness value corresponds to each solution, and, based on this, a list of selected features is provided instead of a single solution. This method performs well on big data and it obtains approximate but acceptable results both in terms of time and in terms of quality, as it returns the near-optimal hyperparameters in less time without using the entire feature set. With regards to optimization methods, the stochastic gradient descent (SGD) and Adam for multiclass and binary classification, respectively, were chosen. Experimentally, one can see that the former is computationally faster while the latter requires less memory. The feature set selection is a difficult task, because the set is specific for each anomaly class and depends on the attack scenario. It serves to eliminate insignificant features and use the relevant ones in training. The results of their work were compared both on multiclass and binary labels, and this approach has a high detection rate with respect to support vector machines (SVM, in Yan and Jia, 2018) in the first case and a similar performance of RF in the second case. In addition, the use of metaheuristics improved anomaly detection compared to a pure application of LSTM. LSTM-RNN-with-GA performs better on large data volumes when comparing its results with those on small datasets.

In Nashville (TN), a city in the USA, 900 million records, such as vehicle speed and traffic jam indicators were classified by Sun et al. (2017) in order to identify traffic anomalies in real time. Two of the open problems in this area are how to synthetically represent data in order to process it without losing useful information and how to augment data with labels useful for supervised machine learning. The crossover operator, used in GA to vary the chromosomes from one species to another, is used in this context to increase the data for training and have balanced classes, while the ROC curve is used to adjust the classifier. In small intervals of time which are assumed to have the same traffic conditions, new data with the same labels are generated. Furthermore, the authors presented an efficient algorithm that converts the available data into traffic condition images (TCI), where each pixel represents a road section and its grayscale value at the average speed therein. Three scenarios were considered to evaluate the impact on traffic and the model used: football matches, hockey matches, and road accidents. Each event has a unique pattern and was used to identify anomalies that deviate from this. A CNN is used which returns a vector in output; it indicates whether an event is recurring traffic or not and the relative time slots, before and after the events, which affect congestion. A random forest was built and tested on the same data, but was surpassed for greater accuracy and lower rate of false anomalies by the CNN. In the hockey game scenario, both methods had more difficulty detecting non-recurring traffic because they have less impact on traffic.

#### 3.1.2. Immune inspired metaheuristics

The human immune system (HIS) is a natural IDS, which distinguishes normal cells from malignant cells and in which killer cells (NK) respond quickly to pathogenic infections by viruses and bacteria. The behavior of the HIS has been modeled to build an artificial immune system applicable to computers. The main characteristics of the immune system are the ability to distinguish self from non-self and the cloning principle. In the artificial immune system research area, the first characteristic has produced many algorithms, denoted as negative selection algorithms (Gupta and Dasgupta, 2022), which have also been applied to the general problem of intrusion detection (Singh et al., 2022) and also anomaly detection (Saurabh and Verma, 2023). The clonal selection principle, likewise, has inspired the design of many algorithms obtained by setting and combining the parameters of the main operators: (i) cloning; (ii) hypermutation; and (iii) aging. In Cutello et al. (2010), the authors studied a large set of numerical functions in order to understand both the search capability and the ability to escape from a local optimal of a clonal selection algorithm.

The detection system called cyber immune system (CIS) designed by Bejoy et al. (2022) was tested on the NSL-KDD dataset, combining the negative selection algorithm with the positive selection algorithm. If the CIS detector identifies a regular traffic pattern, it is removed using negative selection. Using positive selection, random detectors are created to detect personal data, which are saved, otherwise an anomaly is extracted. When the activating receptor of NK cells is activated, they strike; when their inhibitory receptors are activated, they do not. Artificial NK cells likewise spread through the network to detect an intrusion, and are modeled as a non-deterministic finite automaton. For each NK cell an activating range is defined, whereas its fitness defines the probability of proliferation. Each NK cell can be in any six states, denoted as passive, active, activating response, inhibitory response, mature, and cloning, and it has a memory that stores the IDS of detected antigen agents. The higher the fitness value, the lower the mutation rate in the NK cell system. NK cells are initialized to the passive state and when they encounter an abnormal system condition, they switch to the state of activating response. If the NK's fitness value exceeds a certain threshold, its state becomes mature; otherwise, it becomes passive again. Until the maximum NK cell population reaches the mature state, cell proliferation occurs; conversely, the cells revert to the passive state. The threshold values and the maximum population of the algorithm are set by the user. The Euclidean distance or the Hamming distance are chosen to measure the proximity and therefore the affinity between the antigens (anomalies) and the antibodies (artificial NK cells). CIS has a small response time; therefore, it is suitable for real-time intrusion detection and the results have a low false alarm rate.

Hosseini and Seilani (2019) combined negative selection with a ranking algorithm in order to decrease training time and increase its accuracy in the analysis of CICIDS2017 and NSL-KDD datasets. Since network settings are changed periodically by an administrator and new threats can arise, the IDS should detect anomalies in real time. Therefore, in their work, the authors aimed for an updated and improved IDS because it provides two different classes of anomalous and normal data sets in the training phase. Using the Weka tool, the characteristics were selected based on correlation (correlation-based feature selection, CFS), i.e., those that have the highest correlation in the prediction class. As for the negative selection algorithm, they use the cosine similarity formula; automatic training occurs with the five-fold cross validation. The machine learning model is built by an appropriate combination of logistic regression, random forest, K-NN classifier, and decision tree classifier. Furthermore, the use of negative selection makes classification more effective than the use of machine learning algorithms alone, reducing training time for the same performance.

The negative selection algorithm generates detectors and then monitors anomalies, but has the limitation that, on large data sizes, it leads to poor results or an excessive number of detectors. The - distribution estimation-based negative selection algorithm (DENSA) proposed by Fouladvand et al. (2017) has been combined with the Gaussian mixture model (GMM, in Spall and Maryak, 1992) which obtains results in real time and interprets a large amount of data. The parameters of the GMM are determined according to the maximization of the likelihood, through the expectation-maximization algorithm (EM). It is initialized using the k-means clustering algorithm with the Euclidean distance. GMM randomly generates a fixed number of detectors and through an objective function chooses which will be the optimal number of these for a future choice. In fact, the number of detectors must be as small as possible, but in such a way as to cover the entire space. The objective function evaluates the anomaly detection rate and the false positive rate. The proposed algorithm was tested to detect anomalies in archaeological sites in Silakhor, Iran, and also on the NSL-KDD dataset. The results have been compared with that of the V-detector algorithm and those obtained by the proposed method are more accurate.

### 3.2. Swarm intelligence

By swarm intelligence (SI), we mean the collective behavior of decentralized, self-organized systems. There are many models in literature; in all these models, we see that SI systems consist of a population of simple agents which interact with one another and with their environment. Such interactions lead to a global behavior which, externally, appears or is intelligent. Let us briefly describe the two most famous examples:

- The particle swarm optimization (PSO) of (see Kennedy and Eberhart, 1995) reproduces the behavior of swarms of birds and fish. A population of particles, possibly divided into clusters, is the set of candidate solutions. The velocity and the position of each particle are randomly initialized. The algorithm searches for an optimum solution based on fitness value as a measure of quality, weighing particle components such as inertia which affects velocity.
- Ant colony optimization (ACO; see Dorigo et al., 1996) models the behavior and the actions of an ant colony. ACO is a randomized search technique which could be very naturally used in the optimization of paths through graphs.

ACO is a particular example of a general methodology which is denoted as stigmergy (Theraulaz and Bonabeau, 1999). It is a mechanism of indirect coordination and a form of self-organization, through the environment, between agents. The underlying idea is that any trace left in the environment by an individual action has an impact on the actions and performances of other agents. During movement, each individual releases “digital” pheromones similar to those released by ants while searching for food and pheromone traces counteract evaporation when many individuals follow the same path.

Following the work in Gaspar et al. (2021), where four different SI metaheuristics are introduced and compared, we can also mention as examples:

- Ant lion optimization (ALO, in Mirjalili, 2015a) is also inspired by the behavior of ants and the hunting mechanism of the lion ant. The preys communicate with each other through pheromones and the steps of the algorithm include a causal initialization of the population of lion ants and preys, a random walk, a construction of traps, trapping of the preys, reconstruction of the traps, and elitism. For each element of the population, a fitness value is calculated; the population is updated and replaced by choosing the best of it as optimal. The operator called the roulette wheel selects the ants and the lion ant based on their fitness value.
- Artificial bee colonies (ABC, in Garg, 2014) simulates the behavior of onlooker, scout, and employed bees. The search for food resources must be efficient and fast to allow the survival of the colony; therefore, it is necessary find a near-optimal solution in the shortest possible time.
- The bat algorithm (BA; see Yang, 2010) models the behavior of bats, which must distinguish obstacles and prey in the dark, recognizing the various types of insects. Each bat initially moves randomly at a certain speed and with a fixed frequency, subsequently adjusting the wavelength of the sound waves emitted toward the target. The algorithm then updates its position and speed and selects the best solution as the position.

Let us now describe some applications of swarm intelligence metaheuristics to different fields with a particular attention to anomaly detection.

#### 3.2.1. Bee colonies and IDS

In Korczynski et al. (2016), the authors proposed DIAMOND, a fully distributed coordination framework for the cyber defense of a network, inspired by the collaboration of honey bees in search for resources (Karaboga and Akay, 2009). It has among its advantages the ability to find anomalies quickly through a self-organizing system and using little memory. During the collection of nectar and pollen by bees, the constraints of the environment change over time. For the survival of the colony during the winter and reproduction, the bees look for the richest resources and, once they run out of nectar and pollen, they move in search of other resources. Their location and the level of excitement of the bees, corresponding to the abundance of found food, are communicated to their companions through a dance. From the intensity of the signal, the bees decide whether to go to the site and if satisfied, they return to the other bees to recruit them; otherwise, they leave to locate other sites. The same behavior is transposed into DIAMOND for attack detection: sensors cooperate to detect new patterns through their “excitation” about a detected event, without sharing any sensitive information about the attack. During the research process, DIAMOND coordination nodes adjust their sensors autonomously and exchange energy levels called “concern levels” between neighboring nodes, which reflect the perceived probability of network attacks. In addition to the detection units, DIAMOND is equipped with a coordination unit which determines the level of concern, a function of the level of threat it perceives, and the level of concern of the nodes. If enough nodes, also called sensing units, agree to report an anomaly event, it becomes a network attack model. Each detection unit uses its own algorithm for anomaly detection with sensitivity thresholds, which are updated dynamically and independently of the others. The threat level is classified as low (no threat), medium (traffic deviating from the deployment but within the threshold), and high (attack). The detection model combines principal component analysis (PCA), the gamma distribution, and the Kullback-Leibler divergence (Kullback and Leibler, 1951). Furthermore, a limit traffic speed per IP address is considered, above which potential anomalies are reported. Sensitivity and specificity parameters, represented by the percentage of correctly identified malicious packets and that of legitimate packets, are calculated to evaluate the effectiveness of the detection. Comparing these values with those obtained from a (traditional) local reference intrusion detector, there is a 20% improvement and a gain in information starting from when 30% of the nodes coordinate in detecting anomalies.

#### 3.2.2. Metaheuristics vs. fake news

Yildirim (2022) addressed the fake news detection problem through a hybrid multi-thread (HTM) metaheuristic method, in which a supervisor thread (SvT) monitors metaheuristic models that are tested in parallel on different worker threads (WrT). Text on social media is preprocessed by converting all uppercase to lowercase, removing punctuation, and assigning a weight to each word given by the number of repetitions of it present in the news. Only recurring terms with a frequency above a given threshold are chosen to be part of the search space. For each metaheuristic algorithm, the population containing the candidates for that search space is constructed, to which fitness values are assigned for each one. It takes into account the similarity with the text records. At each iteration, the metaheuristic algorithm for searching for fake news is updated by selecting the best solution among the candidates. The best values, gradually obtained by the WrT, are shared on a shared object (ShO) in the multi-thread framework, i.e., SvT and WrT communicate through the ShO. The algorithms applied are PSO, gray wolf optimization (GWO), and dragonfly optimization (DrO) respectively tested on data related to COVID-19 (Koirala, 2021, dataset available in^1^), the Syrian civil war (Salem et al., 2019), and daily politics (Ahmed et al., 2017). Let us briefly describe GWO and DrO.

- GWO (Mirjalili et al., 2014) considers four types of wolves, alpha, beta, delta, and omega, and mimics their hunting behavior consisting of searching, encircling, and attacking the prey. Alpha represents the best candidate for GWO, which however also considers beta and delta successful candidates as second and third place in the Canis lupus hierarchy; omega is the wolf who follows the three leaders. In this case, therefore, for each WrT, the three best candidates obtained in the iterations are compared with those of the ShO. Population candidates are recommended by SvT if better and removed if worse. When one of the thread values comes out better than the three in the ShO, it is added to it and the worst candidate is removed. Conversely, when the WrT contain candidates worse than the ShO, the WrT are updated with a variable better than the ShO.
- DrO (Mirjalili, 2015b) mimics the behaviors of dragonflies in static and dynamic swarms, through different movements within the group such as cohesion, separation, alignment, attraction to the food source, and distraction from enemies. Food and enemies, respectively, represent the best and worst value for the location.

The approach was evaluated on the three datasets and the performances compared with those of 15 known methods. The results showed that HTM is competitive in identifying fake news and works better with PSO, which was faster than GWO, whereas DrO lagged behind the others.

### 3.3. Whale optimization algorithm

We close this section by mentioning another nature-inspired metaheuristic for optimization problems, recently proposed in Mirjalili and Lewis (2016) and denoted as the whale optimization algorithm (WOA). The meta-heuristic is inspired by the special hunting method of humpback whales, called the bubble-net hunting method. Bubbles are created in a spiral pattern as the whale moves to the surface to eat krill and small fish herds. In a WOA, a search agent, which simulates a whale, updates its position in the neighborhood of the current best solution obtained, for the encirclement of the prey. In addition to the bubble method, whales search randomly for prey (exploration phase). WOA algorithms have been shown to have a good balance between exploration and exploitation of the search space, which helps to avoid local optima.

#### 3.3.1. WOA and COVID-19 re-opening policies

A very interesting example of the application of such a metaheuristic to anomaly detection is given by the work in Cuevas et al. (2023). The recent COVID-19 pandemic, aka the coronavirus pandemic, is certainly a good example of anomalies and the WOA metaheuristic is used to support the evaluation of reopening policies and measures so as to minimize the risk of transmission from COVID-19 (anomaly). An effective reopening policy is driven by the whale optimization algorithm integrated with an agent scheme. The collective interaction of these agents on various levels (internal structure, country, city, state) simulates the behavior of the population with a six-rule scheme that controls the risk of infection. In the mentioned work, the authors proposed three scenarios for an optimal evaluation of plausible reopening conditions, under minimal transmission risk, with the agents and their parameters and attributes simulating a hypothetical re-opening context.

Vaccinations, infection, onset of symptoms, incubation, quarantine period, and death are considered, as well as the circumstances under which an agent can become infected. The social behavior of humpback whales through the stages of searching for prey, the bubble-net hunting method, and prey encirclement yields more realistic results than mathematical macro-models. Each solution represents a vector of decision variables, which are modified at each iteration by the WOA operators to obtain the optimal scenario within the stopping criterion. The process to obtain the optimal scenario starts from the initialization of different scenarios, then the infection risk is evaluated through the agent-based model, with each scenario modified according to the operators of the WOA algorithm. In order to determine less restrictive interventions with the lowest risk of infection, the minimum number of vaccinated people inside and the maximum number of people who can be hosted inside a place are estimated, guaranteeing that a low infection rate is still maintained. Determining the number of individuals required to achieve immunity is an optimization problem that balances two important goals: minimizing the number of inoculated people and maximizing the number of people without immunity unimmunized individuals who for either for health reasons or for personal choice are unable to be vaccinated. Furthermore, a relationship is established between the size of a workplace and the maximum capacity as the maximum number of individuals to keep the transmission rate low.

#### 3.3.2. WOA and gene-expression datasets

DNA gene expression datasets are quite significant in the biological research community because they can help in identifying diseases using the so-called “bio-markers” in the gene sequence. Bio-markers are specific alterations or anomalies in the DNA sequence that represent a particular disease. Such anomalies, however, appear in small number, i.e., compared to the gene sequence, only a small number are bio-markers. In Kundu et al. (2022), the authors proposed an improvement of the WOA meta-heuristic, called the altruistic whale optimization algorithm (AltWOA) to efficiently select relevant features (genes) from data for classification. Exhaustively searching for an optimal feature set is an NP-hard problem; therefore, smart approaches are needed to select the optimal subset. The concept of altruism that is being incorporated into the whale population is that some “mediocre” fitness solutions could evolve into promising solutions if allowed to propagate through other iterations. Thus, a more suitable solution might sacrifice itself in favor of one with “potential” (i.e., a solution with more diverse characteristics) and this strategy showed better predictive ability than WOA or other state-of-the-art methods on eight test datasets. Gene expression data, binary class and multiclass, chosen to analyze the performance of the proposed method, were used to detect cancer, leukemia, lymphoma, and other anomalies. Dimensionality reduction techniques and feature selection on a large amount of data have become important tasks nowadays to optimize information storage. In the proposed approach, features with lower entropy values are considered more informative; furthermore, the continuous search of WOA is mapped to the binary search to make the algorithm suitable for the selection of features. To facilitate the choice of solutions, they are grouped in pairs so that each candidate solution is matched to the “most similar” one through a similarity index and the Hamming distance. This metric, given two binary coded candidate solutions, measures the number of places where the elements are different. For each pair, one will be sacrificed for the other based on their generated correlation score. A lower correlation score identifies the chosen solution, because a small score indicates a greater importance of the features, while a high correlation denotes redundancy. The obtained results showed a much more reliable performance of AltWOA compared with 10 other popular feature selection algorithms and its ability to quickly converge (within 10 iterations). While AltWOA takes slightly more computation time than WOA, it performs significantly better.

## 4. Metaheuristic integration to optimize deep networks: a case study

As described in the previous sections, various machine learning techniques are commonly used to detect fast or even in real time any anomaly which could affect the proper functioning of an industrial, automotive, cyber, or space system. Here, we will focus on one particular case study that shows a way to further improve the performance of existing methods by using metaheuristic algorithms.

The integration of machine learning and metaheuristics is a new area that has become increasingly important in recent years. For example, in Kumar Pandey et al. (2022), a genetic algorithm was used to improve the prediction accuracy of a deep network. A tuning of hyperparameters based on metaheuristics was performed and its advantages were discussed. Finally, in Zito et al. (2023a,b), the authors proved that a metaheuristic approach can be used to improve a machine learning model to infer a gene regulatory network from gene expression time series data, which is known to be a non-simple problem.

### 4.1. Designing machine learning architecture

To design a machine learning model, three types of information must be specified: a structure, the parameters of the model (also called *hyperparameters*), and working parameters. In more detail:

- A model structure defines the behavior of the model and must be selected according to the distribution of data to be predicted.
- Hyperparameters control the model and its behavior. They are also independent of the data used to train the model and must be chosen during the design of a model. Examples of hyperparameters include the number of neurons in a fully connected layer, the type of activation function, and the dropout probability in the dropout layer.
- Apart from hyperparameters, working parameters are finally obtained through learning algorithms such as *adaptive moment estimation* (Adam, in Bock and Weiß, 2019) and *stochastic gradient descent* (SGD, in Bottou, 2012). A learning algorithm is used to extrapolate knowledge from the data and use it later to solve a specific task. In our case, such a task is the detection of an anomaly in a system.

Among all the machine learning models presented in the literature, here we consider the architecture of a recurrent neural network as a case study. Basically, a *recurrent neural network* (RNN) is a class of neural networks characterized by the fact that information from past values can be used to compute future values. For this reason, they are used primarily in time series forecasting problems (Hewamalage et al., 2021). Unlike other classes of neural networks, such as *multilayer perceptron* (MLP) and *convolutional neural networks* (CNN), where each layer is stateless, i.e., it computes the output using only the output of the previous layer as input, in a RNN, each layer has an internal state.

**Figure 1:**
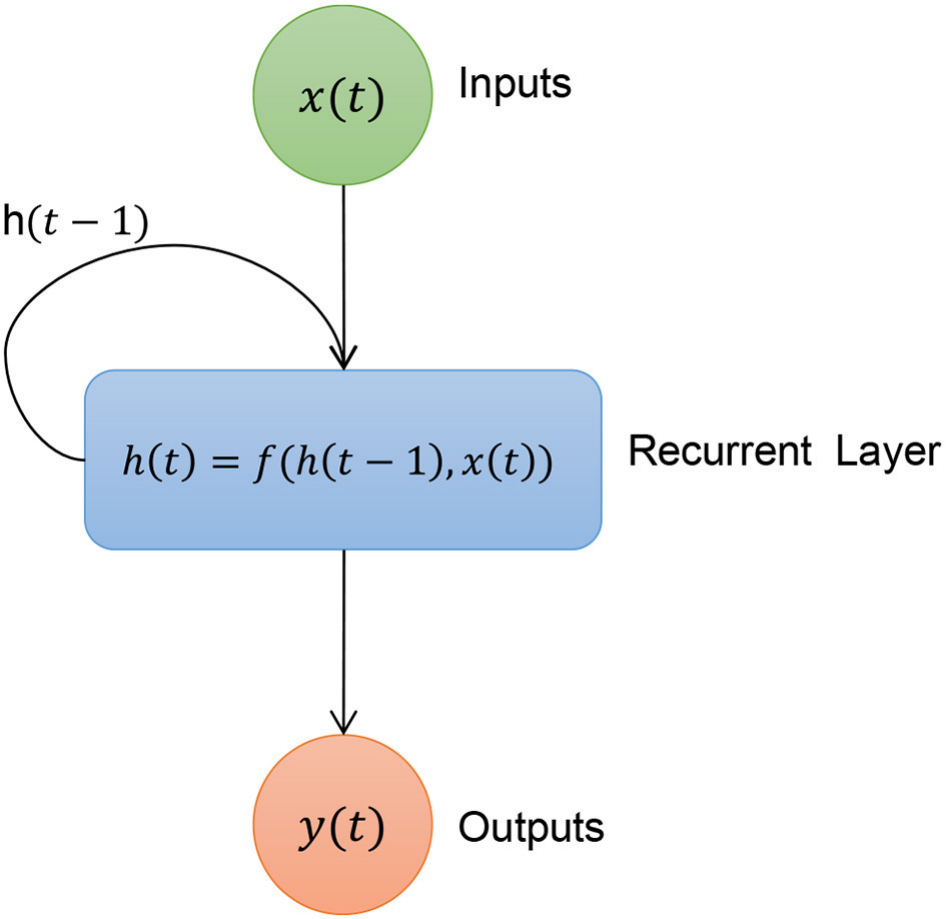
Structure of a recurrent layer.

The output of a recurrent layer is then a combination of the output of the previous layer and its internal state, which is updated based on the type of recurrent layer used. A classic representation of a neural network can be seen in Figure 1. Two recurrent layers most commonly used to implement a recurrent neural network are *long short-term memory* (LSTM) and *gated recurrent units* (GRU, in Shen et al., 2018).

### 4.2. Metaheuristic optimization

The performance of a machine learning model can be enhanced by metaheuristic optimization. Hence, in this subsection we describe how a metaheuristic can be applied to find the optimal combination of hyperparameters for improving the accuracy of a neural network for anomaly detection. For this purpose, in this case study, we employ a *long short-term memory neural network*, and a *genetic algorithm*.

**Figure 2:**
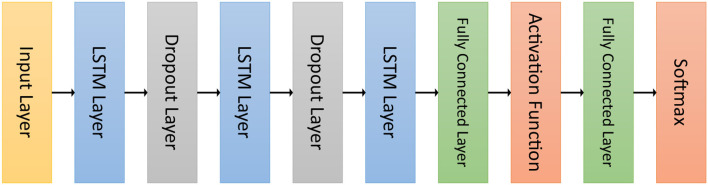
Architecture of the recurrent neural network considered.

Before using a metaheuristic algorithm to optimize its hyperparameters, the architecture of the neural network under consideration must be specified. In this phase, instead of defining a complete neural network with fixed parameters for each layer, we consider a neural network with variable hyperparameters (see Figure 2). The difference between these two types of neural networks is that each solution of the metaheuristic algorithm corresponds to a hyperparameter vector that assigns a value to each parameter of each layer.

Each hyperparameter vector then defines a complete neural network that can be trained and evaluated using a dataset. The evaluation metric used to measure the performance of such a neural network is considered as the fitness function of the solution by the metaheuristic algorithm to find the best hyperparameter vector. At the end of the metaheuristic algorithm, the best hyperparameter vector is returned and used to construct and train the final neural network that is used in practice for this task.

Table 1 shows the range of possible values for each hyperparameter of each type of layer. Only the layers whose hyperparameters are optimized by the metaheuristic algorithm are included in this table. The neural network considered in this study consists of four types of layers: the LSTM layer, dropout layer, fully connected layer, and activation function.

**Table 1:** List of hyperparameters for each type of layer.

**Layer type**	**Symbol**	**Hyperparameter**	**Possible values**
Fully connected layer	FC	Output size	From 10 to 200
Dropout layer	DP	Dropout rate	From 0 to 1
Activation function	AF	Non-linear function name	ReLU, LReLU, ELU, Tanh
LSTM layer	NumUnits	Number of LSTM hidden units	From 50 to 400

### 4.3. Evaluation

We will use the NSL-KDD dataset to train a recurrent neural network for anomaly prediction. The problem we are dealing with can be defined as a classification problem, since we are interested in predicting the class of the ongoing attack. To evaluate the performance of the neural network optimized by a metaheuristic, we need to use metrics that can capture how well it predicts each class and how confident it is about its predictions. Some common metrics for multi-class classification are accuracy, precision, recall, and F1 score.

**Accuracy** is the simplest metric. It measures how many predictions are correct out of all predictions. If we partition the total number of samples *N* into true positive (TP), true negatives (TN), false positive (FP), and false negatives (FN), the accuracy is calculated by simply dividing the sum of the numbers of true positives *TP* and true negatives *TN* by the total number of samples *N*.


(1) $$\begin{array}{l} \text{Accuracy}=\frac{TP+TN}{TP+TN+FP+FN} \end{array}$$

**Precision** is a metric that measures how many positive predictions are actually correct. It tells us how reliable our model is when it predicts a certain class.


(2) $$\begin{array}{l} \text{Precision}=\frac{TP}{TP+FP} \end{array}$$

**Recall** is a metric that measures how many positive samples are correctly predicted. Thus, it tells us how complete our model is when it covers a certain class.


(3) $$\begin{array}{l} \text{Recall}=\frac{TP}{TP+FN} \end{array}$$

**F1 score** is a metric that combines precision and recall into a single value. It is calculated by taking the harmonic mean of precision and recall. F1 score gives us a balanced measure of both reliability and completeness for each class.


(4) $$\begin{array}{l} \text{F1-score}=2\cdot \frac{\text{Precision}\cdot \text{Recall}}{\text{Precision}+\text{Recall}} \end{array}$$

To compute these metrics for multi-class classification, we need to use a *macro-averaging* method that treats each class as an individual binary problem and averages them across all classes (Grandini et al., 2020).

To visually represent the performance of a classification model, a confusion matrix is often employed. Unlike binary classification, in situations where multiple classes are being considered, such as in our case, the confusion matrix will have more than two classes. In the case study analyzed in this section, each class refers a different type of attack. A confusion matrix for multi-class classification is basically a table that is used to evaluate the performance of a machine learning classification model. The confusion matrix is a square matrix with dimensions *M* × *M*, where *M* represents the number of classes. In this particular case, *M* is equal to 5, corresponding to the class of attack under consideration. In more detail, it provides an organized way of mapping predictions to original classes and summarizing correct and incorrect predictions with count values broken down by each class. This allows an easy interpretation and evaluation of a machine learning classification model's performance. In a confusion matrix, each row corresponds to the instances of an actual class and each column corresponds to the instances of a predicted class. The elements along the diagonal indicate the number of instances for which the predicted label matches the true label. Conversely, elements outside the diagonal represent instances that have been misclassified by the classifier. A high value along the diagonal of a confusion matrix is desirable as it denotes a high number of correct predictions.

### 4.4. Results

Let us now show the results obtained by comparing a non-optimized neural network with an optimized neural network. The configurations of the hyperparameters for each layer in a non-optimized neural network were taken from the lstm-nsl-kdd-2022 site,^2^ while the hyperparameters in an optimized neural network were set by using a discrete genetic algorithm (Lin and Hajela, 1992) whose settings are listed in Table 2.

**Table 2:** Parameters used by a genetic algorithm to tune the hyperparameters of a neural network.

**Parameter name**	**Value**
Max iterations	200
Population size	50
Crossover probability	0.6
Mutation probability	0.4
Tournament size	3

**Figure 3:**
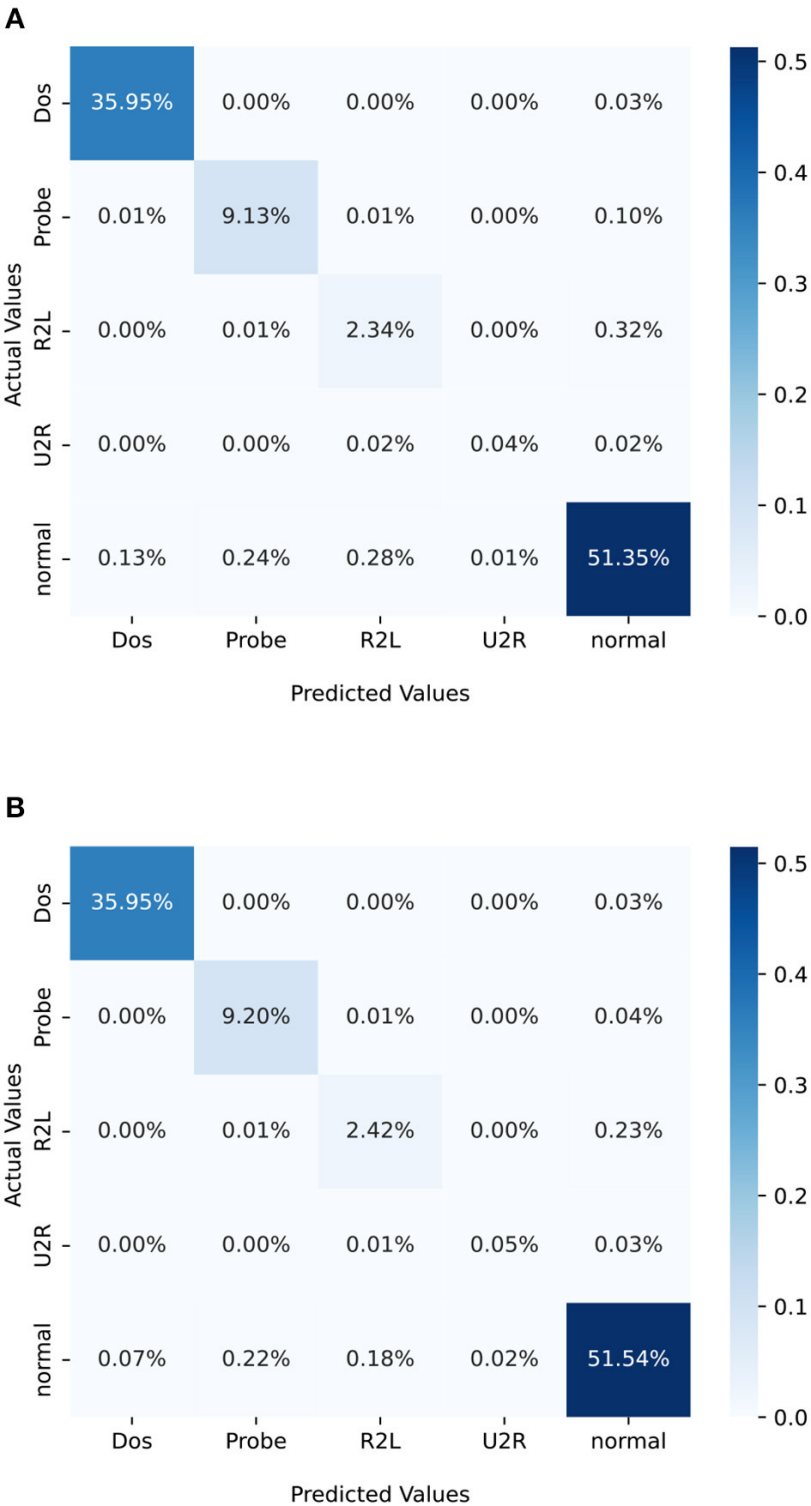
**(A)** Confusion matrix of the non-optimized NN. **(B)** Confusion matrix of the optimized NN.

The performance metrics of a non-optimized neural network and an optimized neural network are presented in Tables 3, 4, respectively. These metrics are based on the evaluation criteria defined in Section 4.3. A comparison of the results reveals that the optimized neural network achieves a higher overall accuracy than the non-optimized one. Moreover, the macro-averaged F1 score, which is a crucial measure for assessing the performance on unbalanced datasets, also shows a slight improvement in the optimized neural network over the non-optimized version. These performance metrics are derived from the two confusion matrices for the optimized and non-optimized neural networks. We express the values as percentages of the total number of observations. Tables 3, 4 show the confusion matrices for the non-optimized network (Figure 3A) and the optimized network (Figure 3B), respectively. In this case, the accuracy of the original neural network is slightly inferior to that of the optimized neural network. This raises the question of whether it is worth the computational effort spent on training the hyperparameter-optimized network. It is important, however, to emphasize that in some applications, such as for instance healthcare or security (as in this case), a small improvement of 1% in accuracy can be considered successful and, as a result, the time taken to train the network becomes irrelevant. The choice of whether or not to optimize hyperparameters depends on the requirements of the application and, therefore, it is important to carefully consider the trade-offs between accuracy and training time when making such decisions.

**Table 3:** Performance metrics of a non-optimized neural network.

**Classes**	**Precision**	**Recall**	**F1 score**
Dos	0.996082	0.999158	0.997618
Probe	0.973099	0.986904	0.979953
R2L	0.886624	0.878788	0.882689
U2R	0.764706	0.520000	0.619048
Normal	0.990970	0.987314	0.989139
**Macro-averaging**	0.922296	0.874433	0.893689
**Accuracy**			0.988251

**Table 4:** Performance metrics of an optimized neural network.

**Classes**	**Precision**	**Recall**	**F1 score**
Dos	0.997944	0.999251	0.998597
Probe	0.975384	0.994543	0.984870
R2L	0.927649	0.906566	0.916986
U2R	0.700000	0.560000	0.622222
Normal	0.993703	0.990809	0.992254
**Macro-averaging**	0.918936	0.890234	0.902986
**Accuracy**			0.991584

## 5. Conclusion and future works

Anomaly detection represents a challenging and important task in many application areas, from industrial cyber-physical systems and related security problems to urban traffic, crowd modeling, social networks, and user privacy. This research work presents a review of several approaches used for anomaly detection, with a main focus on machine learning and metaheuristics. This review highlights how both approaches nowadays have increased their reliability in detection, diagnosis, and in preventing anomalies thanks to their ability to effectively handle and tackle large data. Machine learning methodologies have been commonly used in uncovering anomalies, especially in real time. A hybrid approach for intrusion detection is also presented as a case study. A metaheuristic is integrated within a machine learning model, developing a more robust and efficient solving tool, able to reach high accuracy and precision values. To prove this, the results obtained using the well-known NSL-KDD dataset are presented and described.

The latter approach is part of a new and recent rapidly growing research area, which is the integration of metaheuristics and machine learning. This new hybrid computational method, encouraged by the results presented and included in this paper, may represent an efficient and reliable anomaly detection tool for new future research.

## Author contributions

All authors equally contributed to the development and writing of the paper.

## Footnotes

^1^ https://data.mendeley.com/datasets/zwfdmp5syg/1

^2^ https://www.kaggle.com/code/lionsai/lstm-nsl-kdd-2022
